# Falls From Heights: A Retrospective Review of Roof Fall-Related Trauma

**DOI:** 10.7759/cureus.53727

**Published:** 2024-02-06

**Authors:** Kyle Nugent, Andrew McCague, Austin Henken-Siefken

**Affiliations:** 1 Surgery, Desert Regional Medical Center, Palm Springs, USA; 2 Trauma and Acute Care Surgery, Desert Regional Medical Center, Palm Springs, USA

**Keywords:** heights, roof, fall from height, fall from roof, accidental falls

## Abstract

Background

Falls from roofs (FFRs), while constituting just a segment of all falls, pose a significant public health issue. They not only impact individuals in their daily lives but also pose an increased risk in the construction field. The consequences of these falls range broadly, from minor bruises to serious harm, potentially leading to chronic disability or fatality. For the general populace, such falls might happen during simple activities like maintenance, with outcomes varying from fractures to critical head or spinal injuries. In construction, where elevated work is the norm, the likelihood and potential severity of falls are significantly greater. Construction workers face the threat of falls regularly, with these mishaps often resulting in enduring disabilities that affect both life quality and work capability.

Methodology

This study presents data from a trauma registry system, covering trauma admissions from March 31, 2016, to December 27, 2021, at a level 1 trauma center (Desert Regional Medical Center, Palm Springs, CA). During this five-year and eight-month period, a total of 3,148 patients presented to the emergency department after a fall. This study focuses on patients admitted after an FFR. A retrospective analysis of this data showed that out of these 3,148 patients, 75 presented after an FFR.

Results

In this retrospective analysis of 75 patients presenting to the emergency department after an FFR, the patient profile was predominantly male (70, 93%), with an average age of 51 years. Hospitalization was required for the majority of the patients (70, 93%), with a third necessitating intensive care unit (ICU) care. The necessity for surgical procedures was high at 57 (76%). Upon discharge, 8 (11%) patients were moved to acute rehabilitation. Injuries to the extremities were most common, accounting for 21% (36) of cases, in contrast to facial injuries at 8% (15). Upper extremity fractures were the most prevalent presenting injury at 31% (50), while lower extremity fractures were the least at 6% (9). The overall Injury Severity Score (ISS) averaged 12, with patients having hospital length of stays (LOSs) over 10 days presenting higher ISS scores (18) compared to those with shorter stays (ISS of 11). There was no significant difference in ISS between patients aged 60 and above compared to younger patients.

Conclusions

FFRs represent a substantial cause of injury in both the construction industry and residential settings. This study aims to provide an overview and summary of the existing literature on FFRs, present effective fall prevention methods, and underscore the considerable consequences of such injuries on both construction workers and homeowners. Additionally, it includes an analysis of a dataset detailing injuries resulting from roof-related falls, offering a comparison to existing research.

## Introduction

Falls from roofs (FFRs) present a significant danger, especially in the construction sector, accounting for a third of all fatalities from falls. Alarmingly, about 40% of construction deaths are attributed to FFRs [1]. In the year 2020, there were 108 fatalities among roofing contractors, highlighting the occupational risks [2]. When it comes to residential construction, falls from the edge of the roof are particularly lethal, representing 70% of fall-related work deaths [3]. These figures emphasize the critical need for adequate fall protection, more so because most roofing and ladder accidents happen domestically. The Centers for Disease Control and Prevention (CDC) notes that over 97% of roofing and ladder mishaps occur at home [4], indicating that DIY attempts at installation or repairs can be perilously inadequate. The range of injuries from these accidents is extensive and severe, often including multiple fractures, impalement, and brain and spinal cord trauma, which can lead to long-term disability or fatality [5]. This study aims to provide a summary and analysis of the current research on FFRs and to report the outcomes of FFR injuries using data from a level I trauma center.

## Materials and methods

Our study retrospectively examined de-identified data on trauma admissions from March 31, 2016, to December 27, 2021, at the Desert Regional Medical Center, a level 1 trauma center in Palm Springs, CA. This analysis was concentrated on patients who sustained injuries due to FRFs. We explored various factors such as demographics, the Injury Severity Score (ISS), types of injuries, hospital length of stay (LOS), and the outcomes for these patients. Descriptive statistics were utilized to analyze the data, including median values, standard deviations, and *P*-values to summarize the findings.

## Results

Our dataset from Desert Regional Medical Center, a level 1 trauma center in Palm Springs, CA, documents 75 patients treated for outpatient FFRs from March 31, 2016, to December 27, 2021. Of these patients, 70 (93%) were male and 5 (7%) were female. Elderly patients, defined as those aged 65 and above, numbered 13 (17%). The predominant Hispanic or Latino ethnicity represented 60% (45) of the cohort (Table 1). Of those who presented to the emergency room, 70 (93%) were admitted, with 33 (44%) requiring intensive care unit (ICU) services. Surgical intervention was necessary for 76% (57) of the admitted patients, and 11% (8) were transferred to an acute rehabilitation unit after hospitalization. The study recorded no fatalities. The average LOS in the hospital was 8.3 days, and for the ICU, it was 6.3 days (Table 2). The average age upon admission was 51, in contrast to 45 for those not admitted (Table 3). The overall average ISS was 12, with an ISS of 11 for patients with an LOS of 10 days or fewer, and an ISS of 18 for those with an LOS exceeding 10 days (Table 4). Extremity injuries were most common, occurring in 36 (21%) cases, while facial injuries were least common at 15 (8%) (Table 5). The admissions also included 50 (31%) patients with fractures of the upper limbs, 43 (26%) patients with soft tissue injuries (abrasions, contusions, strains, and sprains), 20 (12%) with hip or femur fractures, 17 (10%) with lacerations, and 9 (6%) with fractures of the lower limbs (Table 6).

**Table 1:** Demographic data. ICU, intensive care unit

Demographics	*n *(%)
Number of patients	75 (100)
Gender	
Male	70 (93)
Female	5 (7)
Age > 65 years	13 (17)
Race	
Hispanic or Latino	45 (60)
Non-Hispanic or Latino	30 (40)
Admissions	70 (93)
ICU admissions	33 (44)

**Table 2:** Admission data. ICU, intensive care unit; LOS, length of stay; SD, standard deviation; ARU, acute rehab unit

Disposition	Number of patients, *n*	Percentage cases (75), %
Admissions	70	93
ICU admissions	33	44
Surgery	57	76
ARU (after discharge)	8	11
Death	0	0
	LOS (days)	SD (days)
Average LOS of admitted patients	8.3	6.92
Average LOS in the ICU	6.3	6.5

**Table 3:** Injury severity by age. SD, standard deviation; ISS, Injury Severity Score

	Average age (years)	SD (years)	P
Admitted	51	17	0.005
Not admitted	45	17	
Age (years)	ISS score	SD (years)	P
≥60	13	8	0.54
<60	12	8	

**Table 4:** Average Injury Severity Score (ISS) by length of stay (LOS). SD, standard deviation

	Average ISS	SD (ISS)	P
ISS score (all patients)	12	8	0.003
ISS score with LOS ≤10 days	11	6	
ISS score with LOS > 10 days	18	9	

**Table 5:** Injury to the body region.

Body region	*n* (%)
Extremity	36 (21)
External	35 (20)
Head	31 (18)
Chest	30 (17)
Abdomen/pelvic	27 (16)
Face	15 (8)

**Table 6:** Presenting injuries on admission. We have categorized abrasions, contusions, strains, and sprains as soft tissue injuries. Additionally, subdural hematomas, subarachnoid hemorrhages, and concussions have been classified as traumatic brain injuries (TBIs).

Injury type	*n* (%)
Upper extremity fracture	50 (31)
Soft tissue injury	43 (26)
TBIs	23 (14)
Hip/femur fracture	20 (12)
Laceration	17 (10)
Lower extremity fracture	9 (6)

## Discussion

It is important to consider the limitations of our research. The small sample size presents a notable constraint, potentially impacting the detection of rarer occurrences or deviations that might be more discernible within a larger sample. Additionally, the absence of detailed information about patients' employment and the precise conditions leading to their injuries limit the generalizability of our findings to other contexts. Furthermore, there is a lack of data on the heights from which patients fell, which limits our understanding of the relationship between fall height and injury severity.

Patient demographics revealed a higher admission rate of males after FFRs, aligning with broader trends observed in research and workplace injury reports. Such trends often highlight that males are more frequently involved in falls from heights, particularly within occupational settings, a pattern largely due to the higher number of males working in high-risk industries like construction [6]. Following their hospitalization, a minimal number of patients required transfer to an acute rehabilitation facility. In contrast, the majority were deemed fit for discharge to their homes or to self-care settings. The observed pattern may be linked to the younger average age of these patients, approximately 51. At this juncture in life, individuals often have a higher potential for self-directed healing from a range of injuries, including bone fractures, skin lacerations, and Traumatic brain injuries (TBIs). Conversely, older adults and young children usually require more extensive care to recover from similar injuries. The recovery duration for these groups tends to be prolonged, especially if there are additional complications or secondary injuries involved.

Our data underscores the significant impact of FFRs on healthcare systems, with a notable number of these incidents resulting in hospitalization and critical care. Although falls are a well-known reason for hospital admissions, there is a lack of specialized research focusing on hospitalizations due to FFRs. Specific findings reveal that over 10 years, 2.3% of trauma admissions at a level 1 trauma center were due to high-level falls [7]. Additionally, falls from 25 feet or higher are linked to increased ISS and longer ICU stays, reflecting the gravity of the injuries sustained which require more intensive medical care [8].

Falls from heights, such as roofs, can result in a range of long-term injuries and conditions that vary in severity based on factors such as the height of the fall, the landing surface, and the physical conditions suffered after the fall. TBIs are among the most serious potential outcomes, with symptoms ranging from mild to severe and affecting cognitive and motor functions [9]. Spinal cord injuries from FFRs can result in different levels of paralysis, and the associated chronic pain and disability can dramatically alter a person's lifestyle and independence. Moreover, the psychological impact of such a traumatic event can be profound, with long-term mental health conditions like post-traumatic stress disorder (PTSD), anxiety, and depression being common among survivors [10]. These conditions highlight the necessity for prompt and extensive medical intervention following FFRs, as well as the potential for enduring health impacts.

Admission data exhibited that the likelihood of hospital admission increased with patient age, with older patients being admitted more frequently than younger ones. Additionally, there's a definitive correlation between higher ISS and extended hospital stays. The ISS is recognized as a dependable indicator of hospital LOS, highlighting a proportional link between the severity of the injury and the time needed for recovery [11]. Considering the greater age of admitted patients often indicates increased frailty and a reduced capacity for recovery, it is anticipated that the average LOS would be longer for these individuals. This is further substantiated by studies that have found that as the ISS rises, which signifies more severe injuries, there is a consequent increase in the duration of hospitalization [12].

The large volume of patient admissions was paralleled by a substantial number requiring surgical procedures. Surgical intervention is frequently necessary for fractures, especially when they are displaced or involve a joint, due to the intricate nature of such injuries and the precision required to realign the bones for effective healing and functionality [13]. In our analysis, fractures to the upper extremities emerged as the predominant type of injury, with these areas being the most commonly affected. This trend can be attributed to the notable incidence of falls on an outstretched hand (FOOSH), particularly from heights. Such injuries usually occur as individuals reflexively extend their hands to cushion the impact of the fall, leading to an increased risk of wrist fractures when falling from over 0.6 m, as the force exceeds the wrist's capacity to absorb the shock without damage [14]. Although our data showed a high rate of upper extremity fractures, this contrasts with other research indicating that falls from elevated places frequently result in significant injuries to the thoracic and lumbar spine, especially around the thoracolumbar junction. These spinal fractures, along with lower limb fractures, constitute a large proportion of injuries in falls from height, as noted in existing literature [15].

Currently, extensive safety protocols are in place to mitigate FFRs within the construction sector. Employers must ensure the provision of fall protection measures such as guardrails, safety nets, or personal fall arrest systems (PFAS) to safeguard their workforce. Occupational Safety and Health Administration (OSHA) mandates that workers on surfaces more than 6 feet above lower levels where the edge is unprotected must be equipped with fall protection [16]. Work performed in proximity to roof edges necessitates additional precautions, including the establishment of controlled access zones and the application of safety system solutions [17] For non-construction workers, such as homeowners engaging in roof work, adopting industry-standard safety practices is crucial. This includes recognizing potential fall hazards, such as skylights or edges without guards, and ensuring the use of sturdy ladders and protective headgear. These strategies are foundational in preventing falls and enhancing safety during roof work. 

## Conclusions

FFRs are a notable cause of injuries, both in the construction sector and domestically. Our research has indicated that individuals most affected by FFRs are typically in their 50s and predominantly male. The injuries sustained often involve the upper extremities and soft tissues, with many cases necessitating surgical intervention. Despite the implementation of safety measures and precautions in environments prone to such accidents, the potential risks associated with FFRs remain significant and warrant continued awareness and preventive strategies.
